# Why we vaccinate incarcerated people first

**DOI:** 10.1016/j.eclinm.2021.100864

**Published:** 2021-05-06

**Authors:** Justin Berk, Josiah D. Rich, Lauren Brinkley-Rubinstein

**Affiliations:** a Rhode Island Department of Health, United States; b Warren Alpert School of Medicine at Brown, United States; c The Center for Health and Justice Transformation at the Miriam, United States; d Medicine and Epidemiology Brown, United States; e University of North Carolina at Chapel Hill School of Medicine, United States

Few settings have been as dramatically affected by the COVID-19 pandemic as jails and prisons across the United States. The majority of the largest national outbreaks of COVID-19 have taken place in carceral facilities with some reporting positive testing rates of over 70%, [1]. The FDA has granted emergency use authorization for two SARS-CoV-2 vaccines that demonstrate near 95% efficiency, offering a life-saving tool to prevent future infections and outbreaks. Vaccine allocation, particularly among incarcerated individuals, remains a controversial topic. Yet there are very clear reasons why incarcerated people should be prioritized for vaccination efforts.

First, incarcerated individuals are at high risk for Covid-19 transmission and severe disease. Congregate living settings, including jails and prisons, facilitate rapid spread of infection. Those in jails and prisons have a 5-fold greater likelihood of infection and 3-fold greater likelihood of death from Covid-19, [2]. Vaccine allocation should follow the epidemiology and be deployed in settings that will have the most impact: where there have been the most cases and more severe disease. Jails and prisons certainly meet these criteria.

Second, jails bring large numbers of individuals into these high-risk spaces and then release them back to the community where the virus can spread to others. Mass incarceration has resulted in nearly 11 million people cycling through the system in any given year, mostly for short-term stays. And, no jail is an island: correctional officers, lawyers, physicians, social workers, dentists, and other staff go in and out of these facilities and ultimately go home to their families in neighboring communities. High rates of infection in a jail can lead to high rates of infection in a community. Indeed, during the first wave of Covid-19 infection, an estimated 16% of state-wide Covid-19 infections were linked to Chicago's Cook County jail alone, [3]. Vaccinating people in jails can serve as an essential part of a multi-pronged public health strategy.

Third, vaccinating incarcerated people can have a substantial impact on Black, Indigenous, and People of Color (BIPOC) communities that have suffered a greater health burden from the pandemic, [4]. Correctional facilities have a disproportionate makeup of these minority groups. High rates of carceral exposure in BIPOC communities further exacerbated COVID-19-related racial disparities, [5]. Moreover, people who have been incarcerated and people who live in communities with a disproportionate burden of incarceration often face other barriers to healthcare access. Many advocates have appropriately argued for vaccine allocation to be equitable. To ensure health equity in vaccine allocation, jails offer a target approach to focus delivery to people of color, of low socioeconomic status, and to marginalized populations that otherwise may not have access to vaccine but suffer a disproportionate burden of disease.

Fourth, vaccinating incarcerated individuals can potentially reduce municipal costs. Most correctional facilities are unable to offer acute level of health care to individuals who become sick and require hospitalization. Covid-19 complications ultimately burden neighboring health systems and hospitals in the area. These costs are ultimately passed on to taxpayers. Preventive vaccination can offer a possible reduction in local or state-level health care expenditures.

Fifth, criminal justice facilities offer one of the most efficient ways to deliver vaccine to a population that has unparalleled accessibility. Many states struggle to efficiently deliver “shots in arms” and have reported backlogs of vaccines that go unused. Jails and prison system, by design, allow easy accessibility to the targeted population. Furthermore, there is some evidence that a substantial proportion of incarcerated people will agree to SARS-CoV-2 vaccination, [6].

Sixth, there is a constitutional duty to provide appropriate health care to those in jail and prison. In the 1976 Supreme Court Case, *Estelle vs. Gamble,* a “deliberate indifference” to the health needs of those incarcerated is a cruel and unusual punishment and thus a violation of the Eight Amendment. Indeed, a federal court in the state of Oregon ruled incarcerated individuals had a constitutional right to vaccine and required prompt distribution to this population, [7].

Seventh, the government has a moral responsibility to provide care to those under the custody of a department of corrections. Their criminal status should not play a role in deciding if they are “worthy” of receiving life-saving care. An individual's sentence was dictated in a court room and did not include contingencies for a global pandemic.

It is for these reasons that the National Academy of Medicine, the American Medical Association, and a consortium of 7 academic institutions have voiced support for the prioritization of this often-overlooked population, [8]. By prioritizing incarcerated people, policymakers can target a high-risk group, decrease community spread, improve equitable allocation, reduce community health system costs, ensure efficient delivery, and align allocation goals with the constitutional and moral duty to care for patients under the government's custody. Certainly, access to vaccine should be available to all as quickly as possible. And while other high-risk groups warrant similar consideration for prioritization, jails and prisons offer a smart public healthy strategy that may otherwise be ignored due to the stigma faced by this marginalized population.

## Declaration of Competing Interest

JR reports receiving grant funding from NIH, NIAID, CFAR Grant # *P30AI042853 and* NIH, NIGMS COBRE Grant #P20GM125507. The other authors have no financial disclosures or conflicts of interest.
